# Therapist Drift from CBT is Associated with Substance Use Among IPV Offenders

**DOI:** 10.1080/10826084.2026.2670618

**Published:** 2026-05-14

**Authors:** Kaitlyn McElroy, Victoria Pezzino, Celeste Sangiorgio, Neha Pawar, Cassandra Berbary, Cory Crane, Caroline J. Easton

**Affiliations:** a Department of Psychology, Oklahoma City University, Oklahoma City, Oklahoma, USA;; b Division of Addiction Psychiatry and Research Program in Psychiatry, University of Rochester Medicine, Rochester, New York, USA;; c Department of Clinical Health Professions, College of Health Science and Technology, Rochester Institute of Technology, Rochester, New York, USA;; d Division of Addiction Psychiatry and Research Program in Psychiatry, University of Rochester Medicine, Rochester, NY 14642, USA

**Keywords:** intimate partner violence, treatment, perpetrators, arrest, fidelity

## Abstract

**Background:**

Alcohol and substance abuse are associated with increased rates of intimate partner violence (IPV). Manualized treatments that target both problems are associated with improved outcomes; however, therapist drift is an understudied factor that may influence outcomes.

**Objective:**

This secondary, observational analysis, part of a National Institute of Health (NIH) funded randomized control study (RCT; NCT07140276), examined associations between therapist drift and treatment outcomes among court-mandated male IPV offenders with alcohol and substance use disorders. Therapist drift was not randomized; therefore, findings should be interpreted as associational rather than causal.

**Methods:**

Sixty-two (*N* = 62) participants were assigned to one of two 12-session treatments: 1) Integrated Cognitive Behavioral Therapy targeting Substance Abuse and Domestic Violence (CBT-SADV) or 2) Drug Counseling (DC) focused on substance use only.

**Results:**

Outcomes included substance use (alcohol and cocaine) and self-reported violence. Analyses included descriptives, logistic regressions, t-tests, and ordinary least squares; due to small sample size and limited therapists, multilevel modeling was not feasible. Therapist drift was high (40.3%) across both treatment conditions and linked to poorer substance use outcomes. Within the SADV condition, higher drift was associated with increased substance use, t(15) = −3.40, *p* = .004. At follow-up, treatment condition was associated with violence related outcomes (*b* = −3.17, *p* = 0.04); but, drift was not uniquely associated with violence.

**Conclusion:**

Findings highlight the importance of maintaining fidelity in manualized treatments to reduce substance use among IPV offenders. Future research should examine these associations in larger, non-court mandated and more diverse samples.

## Therapist drift from CBT is associated with substance use among IPV offenders

Across the United States there were 348,110 victims of intimate partner violence (IPV) in 2022 (Thompson & Tapp, 2022). The experience of IPV poses great individual and societal cost, totaling 110 billion dollars across the lifespans of IPV survivors (Peterson et al., 2018a) and 3.6 trillion in national costs in the form of medical payments, property damage, criminal charges, and losses in productivity (Peterson et al., 2018b). Perpetrators of IPV exhibit common risk factors. Up to 60% of individuals who are violent toward intimate partners use substances including alcohol (Cafferky et al., 2018; Foran & O’Leary, 2008; Gilchrist et al., 2019; Stephens-Lewis et al., 2021) and report high levels of hostility or anger (Norlander & Eckhardt, 2005). Perpetrators of violence are at high risk of IPV during episodes of substance use or withdrawal (Gilchrist et al., 2019).

To address these shared risk factors, treatments of IPV have increasingly integrated Cognitive Behavioral Therapy (CBT) (Snead et al., 2018), which has demonstrated effectiveness in treating aggression and substance use (Easton et al., 2007; Travers et al., 2021). A critical component of treatment fidelity (e.g., adherence and competence) is the treatment provider’s ability to adhere to the skills and principles within these cognitive-behavioral treatment models (Martino et al., 2009; Soleymani et al., 2018). A clinician’s deviation from prescribed CBT content and processes is commonly referred to as therapist drift (Waller & Turner, 2016). While treatment studies for IPV perpetrators often include fidelity monitoring procedures, therapist drift is rarely examined as a *mechanism of treatment outcome* rather than as a manipulation check (Lawson, 2010; Musser et al., 2008).

Use of substances such as cocaine and alcohol represents a particularly high-risk factor for IPV perpetration and is commonly targeted in IPV treatment (Cafferky et al., 2018; Stephens-Lewis et al., 2021). Drug counseling (DC; Mercer & Woody, 1999) targets substance use alone by focusing on psychoeducation, relapse prevention, and recovery skills. However, individuals who engage in IPV consistently report higher levels of anger and hostility compared to those without violent arrest histories (Norlander & Eckhardt, 2005). The Substance Abuse Domestic Violence (SADV) approach is an integrated cognitive-behavioral treatment that targets the relationship between thoughts, emotions, substance use, anger, and aggression (Easton, 2013; Snead et al., 2018).

Despite the availability of empirically supported interventions for substance use disorder (SUD) and IPV, there has been limited examination of the specific mechanisms of behavior change within these treatments. Therapist drift, defined as a failure to deliver prescribed intervention content with adequate adherence, is a critical but understudied aspect of therapist competence (Waller & Turner, 2016). Prior research supports the occurrence of therapist drift in routine care and clinical trials, yet its impact on treatment efficacy remains underexplored (Waller & Turner, 2016; Webb et al., 2010). Cognitive Behavioral Therapy (CBT) research indicates that patients who are assigned and practice CBT coping skills demonstrate significantly better substance use outcomes than those who do not engage in out-of-session practice (Carroll et al., 2005). This underscores the importance of therapist adherence to skill assignment and rehearsal. To date, few studies have examined therapist drift as a continuous, mechanistic predictor of outcomes within integrated IPV-substance use treatment, particularly among court-mandated populations.

The present study is a *secondary, observational analysis* of data from a National Institute of Health (NIH)-funded randomized controlled trial of SADV (Easton, Crane, & Mandel, 2018). Therapist drift was not randomized; therefore, findings are interpreted as *associational rather than causal*. We examined whether therapist drift away from manualized treatment content was associated with substance use and violence outcomes. It was hypothesized that participants whose therapists demonstrated greater drift would exhibit poorer treatment outcomes than participants whose therapists adhered more closely to the manualized intervention. Further, this study highlights what treatment fidelity may look like in clinical practice and how it is implemented across two different manualized treatment interventions. Though both interventions are manualized and highly structured in nature, examining if therapist drift arises and the degree of drift, despite structured check-ins and detailed supervisions, provides insight on the effectiveness of both interventions and their relationship with treatment outcomes in real-world clinical settings. These findings can serve as a first step in identifying if both interventions are appropriate to increasing positive outcomes among a population of individuals in court-mandated therapy who have exposure to IPV and substance use, and if further information can be derived on how to implement and modify interventions and strategies for fidelity in future clinical settings.

## Method

### Sample

A secondary data analysis was conducted using data from the original randomized controlled trial described elsewhere (Easton, Crane, & Mandel, 2018). This analysis focuses specifically on therapist drift as an observational predictor and was not part of the original randomization. The trial was retrospectively registered with ClinicalTrials.gov (NCT07140276) on August 18^th^, 2025.

Participants were recruited from a university-affiliated outpatient substance use treatment program in the Northeastern United States. Eligibility criteria included: a) age 18 or older, b) court-mandated treatment following an IPV charge within the past year; c) reading at or above a fifth-grade level; and d) meeting Diagnostic and Statistical Manual of Mental Disorders-IV (American Psychiatric Association, 1994) criteria for substance use disorder. Exclusion criteria included current psychotic or manic symptoms, suicidal or homicidal ideation, need for acute alcohol detoxification, concurrent substance use or anger treatment, or current psychotropic medication use. Participants were not excluded for use of substances other than alcohol or cocaine; however, analyses focused on alcohol and cocaine due to their strong association with IPV risk. Due to clinic structure, all participants were male.

Seventy-five individuals were screened and oriented toward informed consent approved by the university’s Institutional Review Board. Sixty-two individuals were enrolled and randomized using urn randomization to balance demographic (i.e. age, education, race) and prognostic variables (i.e. number of days of substance use and/or aggressive behavior in the 28 days prior) (Stout et al., 1994).

### Study design

Substance-using participants were randomized to either CBT (treatment focused on addiction and IPV in an integrated treatment approach) or DC (drug counseling that focused only on addiction treatment). Participants received twelve weekly, 60-minute individual therapy sessions. Therapists held master’s degrees and were experienced addiction counselors. Therapists received two days (16 h) of manualized training in their assigned condition and weekly individual supervision. Supervision included review of session content and fidelity checklists; however, therapist drift was not used as a formal corrective variable within supervision.

#### Treatment conditions

Both treatments were manual-guided and were administered to participants in weekly face-to-face individual sessions for approximately 60 min over a 12-week period. Participants in both groups were assigned homework exercises at the end of each session and participants in both groups received positive reinforcement for behavior change and completion of homework/practice exercises.

#### SADV treatment

Substance Abuse Domestic Violence (SADV) is a manualized cognitive-behavioral therapy approach, modified from and used within the Project MATCH manual (Kadden, et al., 1992), focusing on substance use, interpersonal violence, and the relationship between the two. Each session focused on substance use and aggression as the targeted maladaptive behaviors.

Substance Abuse Domestic Violence treatment consisted of 12 Cognitive-Behavioral Therapy sessions designed to reduce co-occurring substance use and IPV. The session topics focused on psychoeducation, communication skills, anger management, and safety planning (Easton et al., 2018). All SADV sessions focused on both substance use and IPV, following the same general format. The first 20 min of each session included a check-in on the participant’s substance use and aggression over the past week and completion of objective measures of substance use with feedback. The next 20 min included a review of the previously assigned homework/practice exercises and continued practice in session. The last 20 min included assigning new homework/practice exercises. For extensive review of the SADV treatment approach see Easton and colleagues (2018). Weekly treatment session content is presented in Table 1.

#### Drug counseling treatment

Drug Counseling is an effective and relevant intervention for substance use. Substance use treatment alone remains an empirically supported strategy to reduce rates of IPV among previously violent samples and was seen as a more ethical control condition than common interventions at the time with no empirical basis, no significant effects, or harmful effects (Eckhardt et al., 2013). Drug Counseling treatment (Mercer & Woody, 1999) consisted of 12 sessions targeting substance use. The sessions focused on topics related to addiction, cravings for substance use, supportive services (e.g., self-help groups), relapse, and recovery. These sessions addressed psychoeducation on addiction, recovery, supportive services, social support, and coping skills for management of negative emotions. Weekly treatment session content is presented in Table 1.

### Measures

#### Therapist drift

Therapist drift was assessed using a 28-item self-report scale on a 7-point scale (1 = not at all, 7 = extensively) on topics covered in sessions adapted from the Yale Adherence and Competence Scale (YACS; Carroll et al., 2000; Corvino et al., 2000). Although therapy sessions were recorded and reviewed in supervision, independent fidelity coding was not conducted for this analysis, and self-report may underestimate drift.

Composite drift scores were calculated across twelve sessions. Reverse-scored treatment-inconsistent behaviors were combined to generate an integrated drift score (α = 0.97). Drift was dichotomized (high vs. low) to address skewed distributions, maintain consistency with the parent trial, and improve interpretability, acknowledging the loss of information inherent in dichotomization.

#### Substance use

Substance use was assessed using urine toxicology (cocaine), breathalyzer testing (alcohol), and Timeline Follow-Back self-report (Marini et al., 2023). A composite substance use variable reflected the presence of any cocaine-positive urine screen or alcohol-positive breathalyzer/self-report across sessions and was dichotomized due to sparse distributions.

#### Self-reported violence

Violence was assessed using the Aggressive Behavior Checklist (Easton et al., 2007). This measure assessed partner-directed violence exclusively, consistent with the IPV focus of the parent trial. Violence variables were dichotomized due to low base rates and judicial monitoring.

### Data analysis

Descriptive statistics characterized the sample. A series of logistic regressions examined associations between therapist drift and outcomes. Analyses were theory-driven and exploratory using a hierarchical block entry rather than stepwise selection. Multilevel modeling was not feasible due to the small sample size and the limited number of therapists. Results should be interpreted cautiously, and future studies should employ multilevel approaches when sample size permits.

## Results

### Participants

Demographic and substance use characteristics are shown in Table 2. Sixty-two participants started treatment. Twenty-nine of these participants were in the SADV condition and thirty-three were in the DC condition. The average age of participants was 39.4 (SD = 8.7), the majority completed a high school education, were employed full time and indicated their racial background as either Caucasian or African American. No pretreatment difference between SADV and DC participants with regards to demographic, substance use, anger, and legal characteristics were noted.

### Scale construction

#### Substance use, self-reported violence and session attendance

Substance use (specifically: urine toxicology for cocaine, and self-reported alcohol use) and violence (specifically: self-reported physical violence, verbal violence, and combined) variables varied in range and means. Variation was reduced by transforming substance use and violence into binary variables where 1 indicated the presence of a behavior and 0 indicated no behavior. The binary variables that we submitted to logistic regression included substance use (1 = positive for cocaine in urine toxicology or alcohol use in breathalyzer or by self-report; 0 = no cocaine or alcohol use identified in urine toxicology or breathalyzer, or self-report respectively), violence (1 = any physical or verbal abuse reported; 0 = no physical or verbal abuse reported), and alcohol use (1 = any self-reported alcohol use; 0 = no self-reported alcohol use). In addition to these variables, we also generated a binary variable for number of sessions attended where 0 = indicated below 9 or fewer sessions and 1 indicated that patients successfully completed a recommended course of treatment with 10 or more sessions attended.

### Therapist drift

Overall, therapists demonstrated a high level of drift, *x* = 5.05 (SD = 0.54). Therapist drift was high across both conditions, with 40.3% of sessions meeting criteria for high therapist drift. In the DC condition, there were a total of 14 participants whose therapists exhibited high drift (42.4%); in the SADV condition, there were a total of 11 participants whose therapists exhibited high drift (37.9%). There was no significant difference in the amount of drift between SADV and DC therapists, t(57.28) = −1.27, *p* = 0.21.

#### Relationships among therapist drift, substance use, and self-reported violence

The relationships among therapist drift and behavioral outcomes were examined through a series of logistic regressions that predicted each behavioral outcome using drift scores. We submitted each dichotomous outcome variable to a stepwise logistic regression, where drift and treatment group were input on the first step, number of sessions was added to the second step, and the interaction between group and drift was added to the third step. Of all these models, only the model predicting substance use was significant, x^2^ (3, *n* = 39) = 8.747, *p* = .03. Therapist drift, treatment group, and the interaction between treatment group and drift significantly predicted substance use (*b* = 4.76, *p* = 0.03, *b* = −21.91, *p* = 0.02, and *b* = 4.75, *p* = 0.03, respectively). Number of sessions approached significance in predicting substance use, *b* = −0.30, *p* = 0.06.

Drift within treatment group was submitted to further analysis using a t-test. There was a nonsignificant relationship between drift and substance use for the drug counseling group; however, for the SADV group, substance use was significantly associated with drift such that higher drift (less adherence to SADV treatment) predicted substance use t(15) = −3.40, *p* = .004. The effect size for this analysis for alcohol specifically was (*d* = 0.85) and was found to exceed Cohen’s (1988) convention for a large effect (*d* = 0.80). The odds ratio (OR) was 4.73. The effect size for the composite variable substance use was (d=−0.0819). The OR was 0.862, indicating that while having a small effect, individuals with a therapist who drifted less from manualized SADV treatment had less substance use.

#### Relationships among therapist drift, substance use, and self-reported violence at follow-up

Self-reported violence was submitted to ordinal least squares regression to examine the effect of therapist drift by treatment groups at follow-up three months following treatment. The model predicting self-reported violence by treatment group and drift was significant, x^2^ (2, 13) = 6.14, *p* = .05. Treatment group was significantly associated with self-reported violence, *b* = −3.17, *p* = 0.04, but the interaction between drift and self-reported violence was not significant in the model. Within-group drift effects for each treatment group were also non-significant in predicting violence and substance use at follow up.

## Discussion

This study examined therapist drift as an associational mechanism linked to substance use outcomes among male IPV perpetrators receiving CBT-based or skills-based treatment. Consistent with prior research, therapist drift was common despite intensive training and supervision (Waller & Turner, 2016). No participants opted out of the current study in favor of a referral to another treatment program and staff were trained in Motivational Interviewing (MI). Of note, the study satisfied treatment mandates, treatment services were free of charge, and compensation was provided for their time. Drift was associated with increased alcohol use among participants receiving SADV but not DC, suggesting that adherence may be particularly critical in complex CBT interventions.

Additionally, this is the first attempt to create a method for evaluating drift but it does tap into previous work by Dr. Kathleen Carroll’s which showed that if therapists did assign CBT coping skill practices to their patients and patients did attempt to do this coping skill, they had significantly better treatment outcomes than those who did not assign/or patients that did not attempt to do the practice skill (Carroll et al., 2005). Cognitive-behavioral therapy skill acquisition depends on structured introduction, rehearsal, and between-session practice. Drift among CBT therapists may disrupt this process, whereas DC’s more didactic structure may be less sensitive to drift. Therapist drift was not associated with violence outcomes, potentially due to judicial monitoring and low base rates. Drift may exert greater influence on proximal factors of IPV, such as substance use, rather than violent behavior itself under legal oversight.

While treatment fidelity is often reported within the course of treatment for IPV offenders (Lawson, 2010; Musser et al., 2008), our findings support that therapist delivery of treatment content is understudied as a contributor to substance use among IPV perpetrators. Substance use is a key contributor to IPV offending (Cafferky et al., 2018; Foran & O’Leary, 2008; Gilchrist et al., 2019; Stephens-Lewis et al., 2021). Further study is needed to examine how therapist adherence to treatment protocols is associated with substance use across different treatments for IPV offending and investigate the relationships among therapist drift, alliance, and competency as interconnected mechanisms to treatment effectiveness in treatment of IPV perpetrators (Campbell et al., 2015; Kazantzis, 2003; Waller & Turner, 2016; Webb et al., 2010).

### Clinical and research implications

Therapist drift was present in both treatment groups, and those who reported higher drift also had higher rates of alcohol use throughout the treatment. When participants reported low drift, they also reported better treatment outcomes. The finding that drift from treatment content contributes to alcohol use, is consistent with findings in treatment studies for individuals enrolled in 12-step facilitation programs (Campbell et al., 2015; Guydish et al., 2014). Overall, these findings have important implications for the training, delivery, and adherence of treatment interventions. Though manualized treatment is highly effective and supported throughout literature, it can be strengthened to improve treatment outcomes for patients. First, this study highlights the importance of minimizing therapist drift, as therapist drift may not be fully preventable despite treatment being provided in highly structured settings with fidelity check-ins, supervision, and standardized two-day training among master’s level clinicians with previous clinical experience. These safeguards are important and necessary for treatment, but additional supports can better enhance adherence among clinicians.

Specifically, for RCT’s, drift may need to be assessed and re-calibrated sooner by providing more intensive supervision strategies to help decrease drift. Although our study used research rigor in measuring treatment fidelity as part of an RCT, many real-world clinics may lack rigor, have shortages of therapists and have high caseloads which likely contributes to increases in therapist drift (Bailey et al., 2021; Waller & Turner, 2016).

Second, it emphasizes the importance of incorporating treatment fidelity assessments or calibration checks into real-world settings to help decrease “therapist drift.” Potential strategies may specifically target training and supervision by remaining highly structured and manualized but also including education on theory or approaches on how to recognize drift and address it in treatment and corrective strategies for the future. Lastly, digital treatments could be used as tools for therapists to help decrease therapist burden by allowing them to use tools that deploy manualized and standardized material that is designed to improve treatment outcomes. Digital tools can be used to augment the therapy experience by providing check-ins within session, reminders or prompts, and interactive feedback and positive reinforcement for patients when completing specific assignments, practicing strategies, or even role-playing (Easton et al., 2021; Loveys et al., 2021; Sangiorgio, 2021).

### Limitations and future directions

This secondary analysis is observational and cannot support causal inference. Due to the limited number of therapists and the exploratory nature of the study, a hierarchical block entry approach focusing on drift, treatment condition, and the interaction between them all was used. Unmeasured therapist-level (experience, caseload, supervision quality) and client-level (baseline severity, alliance) confounders may have influenced results. Outcomes were dichotomized, reducing statistical power. Drift was assessed *via* therapist self-report. The exclusively male, court-mandated sample limits generalizability.

There are several notable limitations to the present study. The current sample consisted of court mandated participants; As previously stated, two thirds of IPV treatment referrals are not court mandated (Karakurt et al., 2019). The current data were drawn from a closely controlled, federally funded randomized clinical trial that was designed to evaluate treatment efficacy. This protocol enforced a rigid protocol for training clinicians and frequently assesses fidelity to treatment. All clinicians had a master’s degree and were provided one hour of supervision weekly. Despite holding a master’s degree, training and experience varied per clinician and data on specific years of DC training and CBT training were not provided. Additionally, clinical hours obtained prior to this placement site were not accounted for indicating some therapists may have more intervention hours compared to others, hence, contributing to drift. Despite these limitations, drift in real-world community clinics would be expected to exceed levels observed in the current study with less frequent and structured fidelity checks and supervision due to natural limitations and barriers to treatment in real-world settings as individuals’ cognitions, emotions, and knowledge can interfere with providing effective treatment. These conditions are more structured compared to standard outpatient community settings and provide a more realistic reflection of integrating a treatment intervention to a population. Future research should include more therapists with specific educational or training requirements to utilize multilevel modeling approaches and address therapist-level variance. It is also important to note the potential bias that may arise with self-report therapist drift versus independent ratings of therapist drift. Despite bias that may arise, self-report measures continue to be an appropriate form of measurement in research, especially when assessing individual’s perceptions and experiences. Future research should incorporate behavioral observation and a standardized fidelity metric in addition to therapist self-report to strengthen validity and confidence in these findings.

Additionally, this sample was exclusively male, reflecting the majority of individuals diverted into treatment for IPV and substance use. Future research may evaluate the relative risk of drift on SADV and DC treatment outcomes among female perpetrators. Further, incorporating additional tools, such as digital tools (DTx CBT), into treatment to minimize drift and standardized treatment when therapists present with varying levels of experience and intervention hours can improve treatment fidelity and outcomes.

In this current study, we had few reports of IPV during the treatment period, which makes it difficult to model variability. Individuals in the present study had active cases that were monitored by the legal system. It is possible that individuals in the present study had less aggression throughout treatment because of the potential for negative consequences if they engaged in aggressive behaviors or used illegal substances. Negative consequences could have included violation of protective orders and incarceration. Another possibility is that therapist drift is particularly impactful to behaviors that increase risk for IPV offending (i.e., substance use; (Cafferky et al., 2018; Gilchrist et al., 2019). Further investigation among individuals who do not have active judicial oversight could help clarify the relationships among therapist drift, violence, and substance use. The effects of drift on effectiveness in the non-criminal justice referred community may differ and should be studied.

Further, addiction severity may potentially impact outcomes attributed to therapist drift. Addiction severity would have been randomly distributed across treatment conditions due to Urn randomization procedures. There is room for future investigation on interactions between severity and drift, such as the possibility that therapists drifted more or less significantly from the treatment protocol if their client experienced greater substance use severity.

Future clinical direction Digital Cognitive-Behavior Therapy (DTx CBT) may be one solution to help clinicians in real world samples, especially when caseloads are large and burn-out rates are high. It allows for distribution of standardized evidence-based CBT skills training while eliminating the possibility of drift through use of virtual avatars, delivering pre-programed/standardized evidenced- based information and skills (Abd-Alrazaq et al., 2020). Patients enrolled in treatment for violence, loneliness, and anxiety and depressive symptoms have endorsed willingness and interest to engage with virtual agents for mental healthcare (Easton et al., 2018; Loveys et al., 2021). A SADV platform has been created for this purpose (Easton et al., 2018, 2021).

Administration of DTx SADV skills training can be a tool for clinicians to deploy standardized doses of evidence-based therapy, while also allowing clinicians time to focus on the working alliance, therefore potentially mediating the effects of drift on treatment. There is evidence that complementary use of DTx tools in traditional psychotherapy for aggressive behavior is associated with effectiveness beyond traditional psychotherapy (Sangiorgio, 2021). Use of DTx in IPV studies may create additional opportunities for assignments and/or rehearsal of key CBT coping skills and other homework exercises, an essential component of CBT effectiveness in treating substance use and IPV offending (Berbary et al., 2018; Carroll et al., 2005).

## Conclusions

Therapist adherence to manualized CBT interventions plays a critical role in reducing substance use among IPV perpetrators. Despite rigorous trial conditions, drift was common and associated with poorer outcomes in CBT- based treatment. Addressing therapist drift through enhanced supervision and scalable digital CBT tools may improve treatment effectiveness, particularly in community settings.

## Figures and Tables

**Table 1.** Description of weekly treatment sessions for SADV and DC.

Session number	CBT (Focus on Addiction and IPV)	DC (Focus Addiction Only)
1	Understanding patterns of substance use and aggression	Introduction to addiction & symptoms of addiction
2	Identifying high risk situations for substance use and aggression	Processes of recovery part I
3	Coping with craving for alcohol use and urges to lose control	Process of recovery part II
4	Problem solving skills related to substance use and conflicts with significant others	Managing cravings: people, places, and things
5	Managing negative mood states with addiction	Relationships in recovery
6	Awareness of anger and use	Self -help groups
7	management of anger related to significant others	Establishing a support system
8	Communication skills training I (nonverbal skills training w/significant others)	Managing feelings in recovery
9	Communication skills training II (verbal skills training with significant others)	Coping with shame and guilt
10	Problem solving skills (e.g., problems related to substance use and IPV)	Warning signs of relapse
11	Coping with criticisms	Maintaining recovery I
12	Emergency planning (triggers for substance use and/or aggression)	Maintaining recovery II

**Table 2.** Demographic, substance use and legal status.

	Mean or %	N	SD
Age	39.31	62	8.76
Ethnicity, in %			
African American	37.1		
European American	46.8		
Latino American	12.9		
Multiracial	1.6		
Did not answer	1.6		
Education in %			
High school or higher	80.6		
Less than high school	19.4		
Single or Divorced	71.0		
Married	29.0		
Employment %			
Full-time	79.0		
Part-time	9.7		
Unemployed	11.3		
Years alcohol use	21.15	60	9.12
Years alcohol misuse	9.94	35	10.20
Years cocaine use	4.5	62	7.81
Arrests for IPV in the last 12 mo %			
Yes	96.7		
No	3.3		
